# Geographic and Sociodemographic Patterns in Prevalence of Diagnosed Diabetes, US, 2021–2024

**DOI:** 10.5888/pcd23.250288

**Published:** 2026-04-09

**Authors:** Nicholas Yell, Michelle Asiedu-Danso, Cassie L. Odahowski, Elizabeth Crouch, Gabriel A. Benavidez

**Affiliations:** 1 Department of Health Services Policy and Management, Arnold School of Public Health, University of South Carolina, Columbia, South Carolina; 2 Department of Public Health, Robbins College of Health and Human Sciences, Baylor University, Waco, Texas; 3 Arnold School of Public Health, University of South Carolina, Columbia, South Carolina

## Abstract

Using pooled data from the 2021–2024 National Health Interview Survey, we compared the prevalence of diagnosed diabetes among rural and urban adults (≥18 y) across multiple sociodemographic factors by using survey-weighted descriptive statistics and *t* tests. Overall prevalence was 9.8%, with higher rates among rural residents (12.3%) than urban residents (9.3%) (*P* < .001); differences persisted across most sociodemographic groups, including by income, education, and race and ethnicity, but were absent among adults aged 65 years or older. The higher prevalence among rural residents compounds existing socioeconomic and racial and ethnic inequities, highlighting the need to monitor both rural–urban and within-rural differences and to prioritize tailored prevention and management strategies.

**Summary**

**What is already known about this topic?**

Diabetes prevalence is higher among population groups who consistently experience social and economic challenges.

**What is added by this report?**

This study provides an updated national analysis of diabetes prevalence, highlighting how rurality interacts with socioeconomic and demographic characteristics in contributing to the burden of diabetes.

**What are the implications for public health practice?**

Tailored prevention and management strategies are needed to address the disproportionate diabetes prevalence in rural communities, particularly among young adults and racial and socioeconomic groups with less access to health care services.

## Objective

As of 2023, 11.3% of the US population had been diagnosed with diabetes (1). Projections suggest that one-third of the US population may be diagnosed with diabetes by 2050 (2). While diabetes is the 7th leading underlying cause of death in the US (3), its true impact on mortality rates is likely undercounted. It contributes significantly to other leading causes of death, such as heart disease, stroke, and kidney disease, making it a major driver of premature death and reduced life expectancy (4).

The prevalence of diabetes is not evenly distributed. Racial and ethnic minority groups, people with low income or educational attainment, and those living in rural areas experience disproportionately higher rates of diabetes and its complications (5,6). These differences reflect broader intersecting structural and geographic patterns in health risk and access to care. However, less is known about the dual effects of rurality and other sociodemographic and economic characteristics and the burden of diabetes throughout the population.

National estimates often mask variation in rural areas, where populations are not homogenous. Single-year survey prevalence estimates often lack sufficient sample sizes to support meaningful rural–urban comparisons, particularly when further stratifying by sociodemographic characteristics. As a result, within-rural differences may be obscured, limiting the ability to tailor rural-specific interventions.

We analyzed national data by rurality status to provide the most current estimates of diabetes prevalence across the US population. These findings can support more precise public health strategies and inform resource allocation efforts that reflect the needs unique to rural and urban populations.

## Methods

This cross-sectional study pooled data from the 2021–2024 National Health Interview Survey (NHIS), a nationally representative, annual household survey conducted by the National Center for Health Statistics (NCHS) to assess the health of noninstitutionalized US residents. The analytic sample comprised adults aged 18 or older who responded to the survey question asking whether a health professional had ever told them that they had diabetes.

The primary outcome was the prevalence of diagnosed diabetes, calculated as the proportion of respondents who reported that a health professional had ever told them that they had diabetes. The main independent variable was rurality, classified according to the NCHS Urban–Rural Classification Scheme for Counties and defined as rural if the respondent lived in a nonmetropolitan area and urban if otherwise (7). Additional covariates were survey year, sex, age group, body mass index (BMI), education level, race and ethnicity, primary insurance type, income-to-poverty ratio, food security status, and self-rated general health.

All analyses incorporated NHIS’s raking-derived weights and accounted for the survey’s complex design. The survey weights were adjusted proportionally to account for the pooled multiyear sample. Data management and analyses were conducted in SAS 9.4 (SAS Institute Inc). Descriptive statistics were used to examine the weighted diagnosed diabetes prevalence across rurality and sociodemographic factors. We used Rao–Scott χ^2^ tests to assess whether rural and urban prevalence rates differed significantly within each subgroup. The degrees of freedom were 1 and the tests were 1-tailed. This study was exempt from institutional review board review because it used only publicly available, de-identified data.

## Results

The overall weighted adult diabetes prevalence was 9.8%, with a higher prevalence among rural residents (12.3%) compared with urban residents (9.3%) (*P* < .001) (Table). This rural–urban difference persisted across most subgroups. Diabetes was more common among men than women in both settings, but the prevalence was higher among rural men (12.7%) and women (12.0%) than it was among their urban counterparts.

**Table:** Diabetes Prevalence, by Rurality and Sociodemographic Characteristics, National Health Interview Survey, 2021–2024

Demographic characteristic	Overall N^a^	Overall diabetes prevalence (95% CI)	Rural N^a^	Rural diabetes prevalence (95% CI)	Urban N^a^	Urban diabetes prevalence (95% CI)	*P* value
Overall	119,160	9.8 (9.5–10.0)	20,393	12.3 (11.6–13.0)	98,767	9.3 (9.1–9.6)	<.001
**Sex**							
Men	54,346	10.2 (9.9–10.5)	9,284	12.7 (11.8–13.6)	45,062	9.8 (9.5–10.1)	<.001
Women	64,798	9.3 (9.0–9.6)	11,109	12.0 (11.1–12.9)	53,689	8.9 (8.6–9.2)	<.001
**Age, y**							
18–24	7,423	1.0 (0.7–1.2)	1,100	1.6 (0.8–2.5)	6,323	0.9 (0.6–1.1)	.03
25–34	17,298	1.7 (1.5–2.0)	2,324	2.5 (1.6–3.5)	14,974	1.6 (1.3–1.9)	.02
35–44	18,609	4.5 (4.1–4.9)	2,523	6.7 (5.4–8.0)	16,086	4.2 (3.8–4.6)	<.001
45–54	16,655	10.0 (9.4–10.6)	2,700	12.6 (10.8–14.3)	13,955	9.6 (9.0–10.2)	<.001
55–64	20,719	15.4 (14.8–16.0)	3,898	17.8 (16.2–19.4)	16,821	15.0 (14.3–15.6)	<.001
≥65	38,198	20.2 (19.6–20.7)	7,823	20.8 (19.7–22.0)	30,375	20.0 (19.4–20.7)	.24
**Body mass index**							
Underweight	1,907	3.2 (2.2–4.2)	314	4.3 (1.6–6.9)	1,593	3.0 (2.0–4.1)	.35
Healthy weight	36,474	4.8 (4.5–5.1)	5,191	5.8 (5.0–6.5)	31,283	4.7 (4.4–4.9)	.003
Overweight	39,904	9.0 (8.7–9.4)	6,552	11.0 (9.9–12.0)	33,352	8.7 (8.4–9.1)	<.001
Obese	38,318	15.5 (15.0–15.9)	7,918	17.7 (16.6–18.8)	30,400	15.0 (14.5–15.5)	<.001
**Education level**							
Less than high school diploma	10,075	16.9 (16.0–17.8)	2,304	17.9 (15.9–20.0)	7,771	16.7 (15.7–17.7)	.28
High school diploma or GED only	30,135	11.3 (10.9–11.7)	7,164	12.9 (12.0–13.9)	22,971	10.9 (10.4–11.4)	<.001
Some college or associate’s degree	33,049	9.8 (9.4–10.1)	6,206	12.0 (11.0–12.9)	26,843	9.4 (9.0–9.8)	<.001
Bachelor’s degree or higher	45,315	6.2 (6.0–6.5)	4,645	8.0 (7.1–8.9)	40,670	6.1 (5.8–6.3)	<.001
**Race and ethnicity**							
Non-Hispanic White	79,080	8.9 (8.6–9.1)	16,764	11.9 (11.2–12.6)	62,316	8.2 (7.9–8.5)	<.001
Non-Hispanic Black	12,768	12.7 (12.0–13.4)	1,477	15.9 (13.5–18.2)	11,291	12.4 (11.7–13.1)	.002
Hispanic	17,196	10.7 (10.1–11.4)	1,148	12.2 (9.4–15.0)	16,048	10.7 (10.0–11.3)	.27
Non-Hispanic other^b^	10,116	10.2 (9.4–10.9)	1,004	14.4 (11.8–17.0)	9,112	9.8 (9.1–10.5)	<.001
**Primary insurance type**							
Public	32,846	16.2 (15.7–16.7)	6,413	17.3 (16.1–18.5)	26,433	16.0 (15.4–16.5)	.048
Private	70,027	7.3 (7.0–7.5)	10,879	9.9 (9.1–10.6)	59,148	6.9 (6.6–7.1)	<.001
Other	7,127	18.1 (17.0–19.2)	1,449	21.7 (19.3–24.1)	5,678	17.4 (16.2–18.6)	.001
Uninsured	8,741	4.9 (4.4–5.4)	1,578	5.4 (4.1–6.8)	7,163	4.8 (4.2–5.3)	.36
**Ratio of family income to FPL**							
<100% of FPL	12,197	13.9 (13.2–14.7)	2,980	16.1 (14.4–17.8)	9,217	13.4 (12.6–14.3)	.004
≥100% of FPL	106,963	9.3 (9.1–9.5)	17,413	11.7 (11.0–12.4)	89,550	8.9 (8.7–9.2)	<.001
**Food security**							
High	98,630	9.1 (8.8–9.3)	16,678	11.4 (10.7–12.1)	81,952	8.7 (8.5–9.0)	<.001
Marginal or less	15,406	13.9 (13.2–14.5)	3,045	17.1 (15.4–18.8)	12,361	13.3 (12.5–14.0)	<.001
**General health status**							
Excellent	24,653	1.4 (1.2–1.5)	3,131	1.8 (1.4–2.3)	21,522	1.3 (1.2–1.5)	.03
Very good	40,347	4.6 (4.4–4.8)	6,285	5.3 (4.8–5.9)	34,062	4.5 (4.2–4.7)	.006
Good	35,344	13.3 (12.9–13.7)	6,610	14.7 (13.6–15.9)	28,734	13.1 (12.6–13.5)	.006
Fair or poor	18,766	27.4 (26.6–28.2)	4,359	28.6 (26.7–30.5)	14,407	27.2 (26.3–28.0)	.17

Abbreviation: FPL, federal poverty level; GED, general equivalency diploma.

a Unweighted frequencies of survey participants in each category.

b Includes non-Hispanic Asian and American Indian populations.

Prevalence increased with age, but rural residents consistently had higher prevalence except among those aged 65 years or older where no rural–urban difference was observed. Diabetes prevalence rose with increasing BMI, and rural residents had higher rates across most BMI categories, except for those who were underweight. Diabetes decreased with higher educational attainment, and rural–urban differences were observed at the high school graduate level and above with rural residents consistently showing higher prevalence. Diabetes was more common among people with low income. Among those with income below the federal poverty level (FPL), rural residents had a higher prevalence (16.1%) than urban residents (13.4%; *P* = .004). This gap persisted among residents at or above the FPL (11.7% rural vs 8.9% urban; *P* < .001). The only insurance category without a rural–urban difference was the uninsured.

Rural–urban differences were observed among non-Hispanic White, Black, and non-Hispanic other populations that included non-Hispanic Asian and American Indian populations with rural residents having a higher prevalence. Differences among Hispanic people were not observed. Diabetes prevalence was higher among those with marginal or lower versus high food security (13.9% vs 9.1%, respectively, overall); within both food-security strata, rural residents had higher prevalences of diabetes than did urban residents: high food security, 11.4% versus 8.7% (*P* < .001); marginal or lower, 17.1% versus 13.3% (*P* < .001).

## Discussion

Using pooled data from 4 years of NHIS, we observed consistently higher diabetes prevalence among rural residents compared with urban residents, a finding that aligns with prior research (8,9). Across both rural and urban populations, diabetes prevalence increased with age. Significant rural–urban differences were observed only for adults aged less than 65 years. Among adults aged 65 or older, prevalence was similar across both groups. Concentration of high diabetes prevalence in working-age adults in rural areas signals future demand for diabetes care as these cohorts age with implications for complications, mortality rates, costs, and strain on rural health systems (10). Tailored prevention efforts and risk-factor control earlier in adulthood, plus stronger rural primary care and care management, are warranted because services, such as diabetes education, are already limited (11).

Diabetes prevalence declined as level of education and income increased. However, rural residents had significantly higher prevalence at nearly every level of education and income, suggesting that rurality amplifies the impact of socioeconomic status, even among those with more resources. Across racial and ethnic groups, rural residents consistently experienced a higher prevalence of diabetes, particularly among non-Hispanic people. Previous research has documented that rural residence can negatively compound the effects of race and socioeconomic status on health outcomes (12).

Although diabetes was more common overall among respondents with marginal or lower food security than those with high food security, the rural–urban gap persisted across both levels with the largest absolute difference observed in the marginal or lower food security group. These findings highlight the potential dual role of food insecurity and rural residence consistent with prior work linking limited food access, care shortages, and structural barriers in rural settings to higher diabetes burden.

This study has limitations, notably the reliance on self-reported data and potential underreporting of diabetes diagnoses. Additionally, our estimates are crude and not age-adjusted, so rural–urban differences may partly reflect underlying age composition. We prioritized crude prevalence as a measure to quantify population burden and avoid instability in small rural subgroups; findings are descriptive and should not be interpreted as age-independent contrasts. Nonetheless, this analysis confirms that rural residents continue to face a disproportionate diabetes burden across most demographic and socioeconomic strata. These findings underscore the need for geographically tailored public health strategies and the importance of tracking not only rural–urban differences but also within-rural variation across sociodemographic groups. This tracking will be increasingly critical as new pharmaceutical interventions, such as glucagon-like peptide-1 (GLP-1) receptor agonists, offer promising opportunities to reduce diabetes incidence. Identifying which populations are being left behind will be essential to ensuring tailored prevention strategies and uniform access to emerging treatments.
